# Protein-Energy Wasting Assessment and Clinical Outcomes in Patients with Acute Kidney Injury: A Systematic Review with Meta-Analysis

**DOI:** 10.3390/nu12092809

**Published:** 2020-09-13

**Authors:** Ban-Hock Khor, Hui-Ci Tiong, Shing Cheng Tan, Raha Abdul Rahman, Abdul Halim Abdul Gafor

**Affiliations:** 1 Department of Medicine, Faculty of Medicine, Universiti Kebangsaan Malaysia, Cheras, Kuala Lumpur 56000, Malaysia; khorbanhock@gmail.com (B.-H.K.); huicitiong@gmail.com (H.-C.T.); 2 UKM Medical Molecular Biology Institute, Universiti Kebangsaan Malaysia, Cheras, Kuala Lumpur 56000, Malaysia; sctan@ukm.edu.my; 3 Department of Anesthesiology and Intensive Care, Faculty of Medicine, Universiti Kebangsaan Malaysia, Cheras, Kuala Lumpur 56000, Malaysia; raha@ppukm.ukm.edu.my

**Keywords:** nutrition assessment, protein-energy wasting, subjective global assessment, acute kidney injury, clinical outcome, systematic review, meta-analysis

## Abstract

Nutritional assessment is essential to identify patients with acute kidney injury (AKI) who are protein-energy wasting (PEW) and at risk of poor clinical outcomes. This systematic review aimed to investigate the relationship of nutritional assessments for PEW with clinical outcomes in patients with AKI. A systematic search was performed in PubMed, Scopus, and Cochrane Library databases using search terms related to PEW, nutrition assessment, and AKI to identify prospective cohort studies that involved AKI adult patients with at least one nutritional assessment performed and reported relevant clinical outcomes, such as mortality, length of stay, and renal outcomes associated with the nutritional parameters. Seventeen studies reporting eight nutritional parameters for PEW assessment were identified and mortality was the main clinical outcome reported. A meta-analysis showed that PEW assessed using subjective global assessment (SGA) was associated with greater mortality risk (RR: 1.99, 95% CI: 1.36–2.91). Individual nutrition parameters, such as serum chemistry, body mass, muscle mass, and dietary intakes, were not consistently associated with mortality. In conclusion, SGA is a valid tool for PEW assessment in patients with AKI, while other nutrition parameters in isolation had limited validity for PEW assessment.

## 1. Introduction

Acute kidney injury (AKI) is a heterogeneous group of syndromes defined by a sudden decline in glomerular filtration rate followed by an increase in serum creatinine or oliguria [1]. There are several definitions and staging systems for AKI, namely Kidney Disease Improving Global Outcomes (KDIGO) [2], Acute Kidney Injury Network [3], and Risk, Injury, Failure, Loss, and End-Stage Kidney Disease (RIFLE) [4]. The prevalence of AKI ranged from 3% to 18.3% in hospitalized adult patients and 33% to 66.7% in critically ill patients. It is estimated that less than 10% non-critically ill hospitalized patients with AKI will require kidney replacement therapy (KRT) while about 5% to 11% critically ill adults with AKI will require KRT [5]. An episode of AKI is associated with increased risk of mortality as well as long-term adverse outcomes such as new onset or worsening of chronic kidney disease (CKD), dialysis dependence, and cardiovascular disease [5,6].

Protein-energy wasting (PEW) was proposed by the International Society of Renal Nutrition and Metabolism (ISRNM) to define a state of decreased body stores of protein and energy fuels (body protein and fat masses) in patients with CKD and AKI [7]. PEW is prevalent in AKI patients and a meta-analysis of two studies reported that the prevalence of PEW in patients with AKI was 66.7% [8]. The pathogenesis of PEW in AKI is multifactorial, including metabolic alterations and impaired homeostasis responses due to sudden loss of kidney function [9], intrarenal and systemic inflammation associated with AKI [10], hypercatabolic state from the underlying comorbidity and critical illness [11], and amino acid loss in KRTs [12].

Nutritional status assessment is critical to identify patients who are PEW and at risk of mortality. The ISRNM expert panel recommends that clinical diagnosis of PEW in CKD and AKI requires at least three out of four main categories, namely biochemical criteria; low body weight, reduced total body fat, or weight loss; a decrease in muscle mass; low protein or energy intakes [7]. Subjective global assessment (SGA) has also been used for assessment of PEW [8]. These nutrition parameters have been widely used for diagnosis of PEW in patients with CKD as the relationship between these parameters and adverse outcomes in CKD is well established [13]. However, it should be noted that the context of PEW in patients with acute illness might differ from patients with chronic diseases. Therefore, the applicability and reliability of these criteria for nutritional status evaluation in AKI patients, who are often presented with fluid accumulation and deranged metabolic profile, remains unclear [14,15]. In addition, the predictive validity of these standard nutrition parameters in patients with AKI has yet to be reviewed. Therefore, the present systematic review aimed to examine the relationship of PEW and individual nutrition parameter of PEW with clinical outcomes in patients with AKI.

## 2. Methods

### 2.1. Study Protocol

This systematic review was conducted according to the Preferred Reporting Items for Systematic Reviews and Meta-Analyses [16], and the protocol of this systematic review was registered on the International Prospective Register of Systematic Review (CRD42020168595).

### 2.2. Search Strategy

We performed a comprehensive search in PubMed, Scopus, and Cochrane Central Register of Controlled Trials (CENTRAL) on 12 February 2020 to identify relevant studies. Medical Subject Headings terms and free-text terms for AKI and PEW were used as the search strategy (Table S1). We also included *nutrition assessment*, *malnutrition*, and *protein-energy malnutrition* in our search strategy, as these terms were commonly used before the introduction of PEW in 2008 [7]. In addition, we manually search for eligible studies by checking the reference lists of relevant original articles, reviews, and guidelines from inception to February 2020. Our research was restricted to English-language studies, as it has been shown that exclusion of non-English literature does not introduce systematic bias [17].

### 2.3. Study Inclusion and Exclusion Criteria

Prospective cohort studies were considered eligible and included in this review if they reported the association between at least one of the readily utilizable nutritional parameter for PEW assessment (Table S2) [7] and an outcome of interest in adult patients (≥18 years old) diagnosed with AKI. Briefly, these parameters included assessment of nutritional biochemistry, body mass, muscle mass, and dietary intake. We also included studies applying SGA for PEW diagnosis, because this nutritional assessment score was validated, well-established, and had been used for PEW diagnosis in patients with AKI [8]. However, potential tools for PEW assessment such as appetite, energy expenditure, or inflammatory markers were not included in the present review as there were no definite cut-offs for these measures to diagnosis PEW [7]. The outcome measures included mortality (short-term and long-term), hospital or intensive care unit (ICU) length of stay, or renal related outcomes such as KRT dependence, KRT duration, KRT-free days, and renal recovery (either complete, partial, or absent). There was no restriction on the sample size and length of follow-up. We excluded retrospective studies due to potential bias and residual confounding. Studies involving pediatric populations, solid organ transplant recipients, patients with hepatorenal or cardiorenal syndromes, and patients with pregnancy-associated kidney disease were also excluded. In addition, we excluded studies with only univariate or receiver operating characteristic curve analyses, as other non-nutritional factors influencing the clinical outcomes should be fully adjusted in the statistical model.

### 2.4. Study Selection and Data Extraction

Citations from the initial search results of each database were exported to EndNote (version X7.5.3), and duplicates were removed. Two authors (B.-H.K. and H.-C.T.) screened and reviewed the titles and abstracts. Then, full texts of potential studies were retrieved and independently reviewed in details for inclusion based on pre-determined criteria. Discrepancies between two authors were resolved by discussion, and a third author (A.H.A.G.) was referred if consensus could not be reached.

One author (H.-C.T.) extracted the data from included study into a piloted sheet and another author (B.-H.K) crosschecked the extracted data. Disagreements were resolved by discussion, and a third author (A.H.A.G.) was consulted if necessary. The following data were extracted: study characteristics (country, sample size, and patient population), patients’ characteristics (age, sex, comorbidities, primary cause of AKI, requirement of KRT, type of KRT, and disease severity score), nutrition assessment for PEW, multivariable-adjusted risk estimates of clinical outcomes associated with PEW parameters such as beta coefficient (β), odds ratio (OR), hazard ratio (HR), risk ratio (RR) with their corresponding 95% confidence interval (95% CI) and/or *p*-value. 

### 2.5. Assessment of Risk of Bias

Two authors (B.-H.K. and H.-C.T.) independently performed the study quality and risk of bias assessment using the Newcastle-Ottawa Scale [18]. A “star system” was used to appraise a study based on three perspectives, selection of the study cohort, comparability of the cohort, and clarity of outcome assessment and completeness of follow up. A maximum of nine stars can be given for one study.

### 2.6. Statistical Analysis

A meta-analysis was performed to determine the association between PEW (SGA class B and C) and mortality. The pooled risk ratio and 95% CI was calculated based on the sample size and risk ratio of each study. Heterogeneity among the studies was assessed using the chi-squared and *I*^2^ statistics, whereby a *p* value < 0.1 and *I*^2^ above 50% was considered having significant heterogeneity and a random effect model was used. A sensitivity analysis was performed by sequentially omitting one study at a time to verify that the overall result was not influenced by any single study. Publication bias was assessed by using Begg’s and Egger’s tests, as well as by visually inspecting the symmetry of the funnel plot. STATA software (version 16.0, StataCorp, College Station, TX, USA) was used for the analysis.

## 3. Results

### 3.1. Study Characteristics

**Figure 1:**
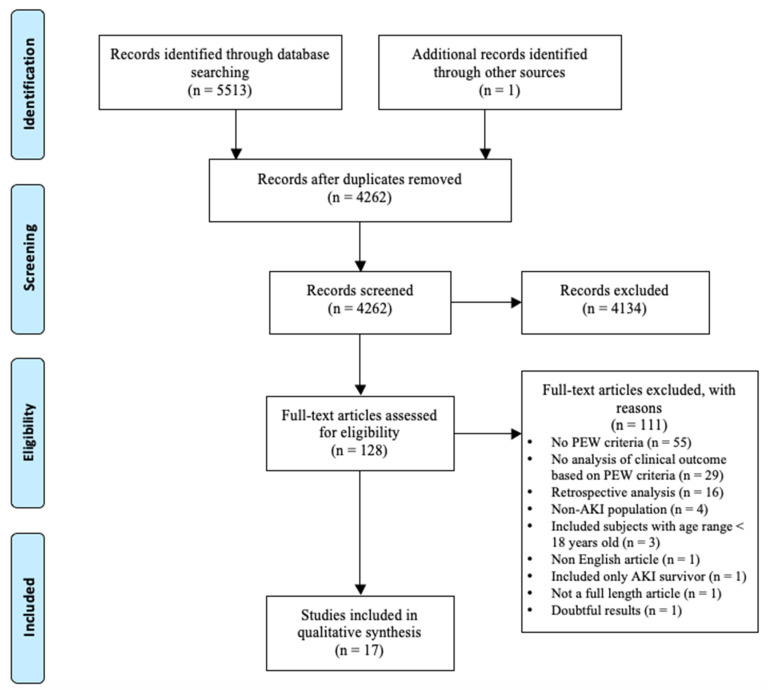
Preferred reporting items for systematic reviews and meta-analyses study flow for literature search and study selection process. Abbreviations: AKI, acute kidney injury; PEW, protein-energy wasting.

The flowchart of study selection is presented in Figure 1. From the literature search, 4262 unique citations were identified from three databases. After screening the title and abstract, 128 studies were retrieved for full-text review. From this, 111 studies were excluded, and the reasons for exclusion are presented in Table S3. Out of the 17 studies included in the present systematic review [19,20,21,22,23,24,25,26,27,28,29,30,31,32,33,34,35] (Table 1 and Table S4), two studies involved patients from the same cohort [19,20], but both studies were included because different nutritional parameters were reported. 

Most studies (*n* = 11) recruited critically ill patients with AKI; two studies each recruited patients with hospitalized-acquired AKI and patients with AKI secondary to ATN, respectively; and one study each recruited elderly patients with AKI and patients from the renal intermediate care unit, respectively. The sample size ranged from 56 to 1457 patients, with only seven studies having sample size more than 200 patients. The mean or median age ranged from 58.1 to 77.9 years and 53.1 to 74.2% were male patients. The mean or median serum creatinine ranged from 158 to 522 μmol/L with three studies [27,34,35] had relatively lower serum creatinine (158–165 μmol/L), as the RIFLE criteria was used for diagnosis of AKI. All patients from six studies were receiving KRT, while the percentage of patients on KRT for 11 studies ranged from 12.1% to 67%. Patients with sepsis ranged from 17.1 to 64.3% and the mean or median C-reactive protein reported ranging from 6.6 to 26.5 mg/dL (Table S4).

For the PEW parameter, most of the studies (*n* = 11) had assessed serum chemistry, mainly serum albumin (*n* = 8), followed by serum total cholesterol (*n* = 4), and serum pre-albumin (*n* = 3). One study each had assessed the body mass index (BMI) for the body mass category and arm circumference for the muscle mass category, respectively. Six studies had evaluated dietary intake, with three studies reporting energy intake and four studies reporting protein intake. There were four studies applying SGA for diagnosis of PEW.

All studies reported mortality as the clinical outcome, while only two studies reported other clinical outcomes such as KRT-free day, ICU-free day, and hospital-free day. Five studies each reported 28-day mortality and 90-day mortality, respectively; four studies reported in-hospital mortality; and two studies each reported overall mortality and 60-day mortality, respectively. The median rate of mortality for all studies was 48% (range, 27.6 to 73.0%). In particular, the 28-, 60-, and 90-day mortality ranged from 61.4 to 73.0%, 36.0 to 50.0%, and 27.6 to 59.9%, respectively, while the in-hospital mortality ranged from 39.0 to 53.0%. 

### 3.2. Nutritional Assessment of PEW and Clinical Outcomes

The relationships between nutritional assessments of PEW and clinical outcomes in patients with AKI are summarized in Table 2. The mean or median of serum albumin reported was ranging from 2.4 to 3.4 g/dL. Two studies showed that serum albumin was not associated with either mortality [33] or in-hospital mortality [21], whilst another two studies reported otherwise [22,31]. Contradictory findings on the association between serum albumin and 60-day mortality were noted in two studies [23,32], while one study reported a significant association between serum albumin and 28-day mortality [25]. Another study showed that serum albumin (per 0.5 g/dL decrease) was not associated with 90-day mortality [35]. For serum pre-albumin, a mean or median ranging from 13.5 to 17.6 mg/dL was reported by three studies. Gong et al. [27] showed that serum pre-albumin was not associated with mortality in elderly patients with AKI. Similarly, Xie et al. [35] also reported that serum pre-albumin (per 5 mg/dL decrease) was not associated with 90-day mortality. In contrast, Wang et al. [34] found that AKI patients with serum pre-albumin <10 mg/dL had 2.55 times increased HR for 90-day mortality. For serum total cholesterol, the mean or median reported by four studies ranged from 101 to 139 mg/dL. Two studies found no significant association between serum total cholesterol and in-hospital mortality [21,22]. Similarly, Xie et al. [35] reported that serum total cholesterol (per 3 mg/dL decrease) was not associated with 90-day mortality. Contrarily, Guimaraes et al. [28] showed that serum total cholesterol ≤96 mg/dL was associated with 28-day mortality (HR: 10.94, 95% CI: 1.89–63.29, *p* = 0.008).

For the body mass category, Lin et al. [30] showed that BMI was associated with mortality (OR: 0.903, 95% CI 0.840–0.971, *p* = 0.006) in patients with post-operative AKI receiving KRT. For the muscle mass category, one study reported that arm circumference was not associated with in-hospital mortality (OR: 0.961; 95% CI: 0.850–1.086, *p* = 0.52) [21].

The means of energy and protein intake ranged from 11.0 to 13.5 kcal/kg and 0.50 to 0.64 g/kg, respectively. Bellomo et al. [19,20] reported that neither energy nor protein intake was associated with 90-day mortality, KRT-free days, mechanical ventilation-free day, ICU-free days, and hospital-free day. Energy intake >25 kcal/kg/day was not associated with 28-day mortality or ICU discharge. Similarly, protein intake >1 g/kg/day was not also associated with mortality. Contrarily, two studies showed that energy intake was associated with ~5% lower risk of in-hospital mortality [21,22]. As for dietary protein intake, one study each found that protein intake was associated lower risk of in-hospital mortality (OR: 0.947; 95% CI: 0.988–0.992; *p* = 0.028) [22] and 28-day mortality (HR: 0.993, 95% CI: 0.987–0.999, *p* = 0.032) [24], respectively. However, it is important to note that the upper limits of 95% CI of these studies [21,22,24] were very close to 1.00, therefore, the clinical significance remains unclear. Kritmetapak et al. [29] also showed that dietary protein (per 0.2 g/kg increase) was associated with survival on 28th day (OR: 4.62; 95% CI: 1.48–14.47; *p* = 0.009). It is also worth highlighting that the analyses by Bellomo et al. [19,20] included adjustment with more prognostic covariates, whereas the statistical analyses of other studies [22,24,29] were considered minimally adjusted.

**Figure 2:**
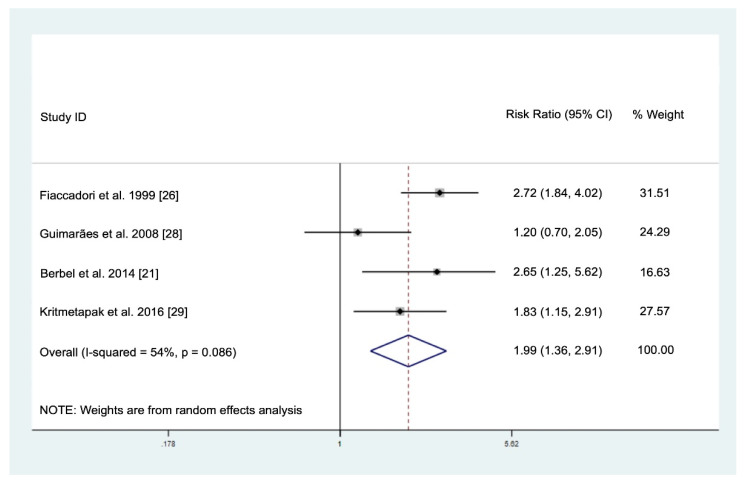
Pooled risk ratio for the association of protein-energy wasting with mortality in patients with acute kidney injury.

There were four studies reporting the prevalence of PEW based on SGA in patients with AKI (Table 3). The prevalence of severely malnourished patients was highest (41.7%) in the intermediate care unit [26], while 41.7–68.8% patients from ICU and/or wards were mildly/moderately malnourished [21,28,29]. The meta-analysis of these four studies showed that PEW (SGA class B or C) was associated with increased mortality (RR: 1.99, 95% CI: 1.36–2.91) in patients with AKI (Figure 2). The sensitivity analysis showed that exclusion of each of the studies did not affect the overall direction and magnitude of the result, suggesting that the result was stable and not driven by any single study (Figure S1). The Egger’s test (*p* = 0.748) and Begg’s test (*p* = 0.497) as well as visual inspection of the funnel plot (Figure S2) showed no evidence of publication bias.

### 3.3. Quality Assessment

Based on the Newcastle-Ottawa Scale assessment, six studies were rated with 9 stars, three studies were rated with 8 stars, five studies were rated with 7 stars, two studies were rated with 6 stars, and one study was rated with 5 stars (Table S5). Nine studies (52.9%) rated with 8 or 9 stars were considered as a low risk of bias. Majority of the studies did not report the detailed process of patient recruitment and duration or completeness of follow-up. In addition, some studies had performed minimal statistical adjustments for the predictive validity analyses.

## 4. Discussion

This systematic review set out with the aim to examine the association between nutritional assessments of PEW and clinical outcomes in patients with AKI. We observed a high prevalence of PEW (ranging from 67.9 to 82.1%) assessed via SGA, and PEW was associated with almost two times greater risk of mortality. However, our literature search identified no study applying the ISRNM criteria for PEW diagnosis in patients with AKI, which could be related to the limited feasibility and applicability. As we assessed each individual nutritional parameter of PEW, there was inconsistent evidence on the associations between these parameters and clinical outcomes in patients with AKI. The PEW criteria proposed by the ISRNM are mainly derived from data obtained in patients with CKD. Therefore, the cut-off values may not be relevant for patients with AKI experiencing drastic deterioration in nutritional status. Further validation for the PEW diagnostic criteria by the ISRNM in patients with AKI remains necessary.

Serum albumin was the most frequently reported nutritional biomarker, as it is relatively inexpensive and widely available, though serum pre-albumin is considered more sensitive due to its shorter half-life (2–3 days) than serum albumin (20 days) [37]. Although the synthesis of albumin and pre-albumin is influenced by dietary protein intakes, non-nutritional factors such as inflammation, overhydration, physiological stress, and infection are also implicated in low levels of serum albumin or pre-albumin [37]. Epidemiological studies have consistently shown that low levels of serum albumin and pre-albumin are strongly associated with poor clinical outcomes in dialysis patients [7,38,39]. However, patients with AKI are generally presented with a more profound hypoalbuminemia or hypoprealbuminemia compared to CKD patients on dialysis, due to a greater degree of underlying inflammatory response during acute illness. In the present review, it was observed that the mean or median serum albumin levels of patients with AKI were much lower than normal values, which was attributed to sepsis and accompanying systemic inflammation. During acute illness, the distribution of serum albumin between the intravascular and extravascular compartments is altered due to increased capillary leakage. In addition, the rate of albumin synthesis is reduced, while the albumin degradation rate is increased in the presence of critical illness [40]. Therefore, hypoalbuminemia is prevalent at the early phase of acute illness and its concentration will only increase during the recovery phase. Although a meta-analysis by Wiedermann [41] showed that hypoalbuminemia is a significant predictor of mortality in patients with AKI, this meta-analysis included studies up to year 2009 only and was limited by inclusion of studies with retrospective design. Serum pre-albumin below 30 mg/dL is indicative as PEW in dialysis patients [7]; Wang et al. [34] showed that only AKI patients with a serum pre-albumin level below 10 mg/dL had a significantly higher 90-day mortality risk. Serum albumin and pre-albumin level in patients with AKI, particularly those with critical illness may reflect the severity of illness instead of caloric and protein deficits [42]. Therefore, these biomarkers alone are not reliable in diagnosing PEW in AKI patients. In fact, serum albumin and pre-albumin are not included in the standardized diagnostic criteria for malnutrition proposed by the Academy of Nutrition and Dietetics/American Society for Parenteral and Enteral Nutrition [43], while the Global Leadership Initiative Malnutrition suggests that serum albumin and pre-albumin may be used as a supportive proxy measure of inflammation [44].

Serum cholesterol is a parameter of malnutrition that has been included in several nutritional screening and assessment tools [36]. A meta-analysis demonstrated that serum cholesterol was associated with the risk of malnutrition in older adults with or without acute diseases [45]. Hypocholesterolemia is paradoxically associated with reduced risk of mortality in patients on maintenance hemodialysis [46], attributed to the cholesterol-lowering effect of systemic inflammation and malnutrition [47]. Similar to other visceral proteins, hypocholesterolemia had been documented in critically injured patients, which was correlated with organ dysfunction and presence of infections [48]. 

Evaluation of body mass and muscle mass using anthropometric measures and physical examination are important diagnostic criteria of PEW [7]. BMI is the most widely used indicator of body mass and has been shown to be paradoxically associated with mortality in CKD patients on dialysis [49]. The obesity paradox observed in patients with renal insufficiency may be explained by higher plasma levels of protective mediators, a greater hemodynamic stability during KRT, and adipose tissue acting as a “buffer” for uremic toxins [50]. Lin et al. [30] reported that BMI was significantly associated with lower 90-day mortality in patients with postoperative AKI in ICU. It should be noted that this population had a relatively normal mean BMI (~23.5 kg/m^2^), therefore this finding should be interpreted as potential harm associated with lower BMI rather than survival benefit of obesity. Schiffl [51] reviewed eight retrospective studies, which showed conflicting findings on the association between BMI and mortality in critically ill patients with AKI, and the author suggested that the obesity paradox observed in patients with AKI could be attributed to statistical fallacy and the result of chance, bias, and residual confounding variables in retrospective cohort analyses. Essentially, PEW occurs at any BMI and unintentional weight loss should also be considered as an important indicator of PEW [7]. In the context of acute illness, unintentional weight loss of more than 1% within a week should be suggestive of PEW [43], but assessment of weight loss in patients with AKI is challenging as weight changes are often masked with fluid retention. 

Patients with AKI experience major metabolic alterations, which lead to muscle wasting, a fundamental feature of PEW. Objective and feasible assessment of muscle mass is essential for diagnosis of PEW. Mid-arm muscle circumference is a measured surrogate of lean body mass that has been shown to be a significant predictor of survival in patients on maintenance hemodialysis [52]. However, Berbel et al. [21] reported that arm muscle circumference was not associated with mortality in patients with AKI. Measurement of arm circumference is not sensitive to identify muscle wasting during acute illness [53], and the accuracy and practicability of this anthropometric measurement in sedated or bed-ridden patients with edema is uncertain. Sabatino et al. [54] proposed that ultrasound imaging of quadriceps muscle is a reliable and non-invasive method for evaluation of skeletal muscle in patients with AKI, and this method had been validated against the computerized tomography scan [55]. Further studies investigating the potential utility of this muscle assessment method in patients with AKI are warranted.

Suboptimal nutritional intake is one of the contributory factors to development of PEW. The nutritional requirements of patients with AKI are affected by disease severity, pre-existing nutritional status, underlying co-morbidities, and KRT modality. The clinical practice guidelines have recommended energy and protein intakes of 20–30 kcal/kg/day and 1.2–2.0 g/kg/day for patients with AKI, respectively [2,56]. However, the present review showed that the means of energy and protein intake among patients with AKI were much lower than the recommended amount [19,20,21,22,24,29]. There was inconclusive evidence on the association between energy and protein intake with clinical outcomes in patients with AKI, and those studies that reported higher mortality risk in patients with low energy and protein intakes had limitations with the statistical models that were not fully adjusted. Meta-analyses of randomized controlled trials have demonstrated that energy and protein provisions were not associated with clinical outcomes in critically ill patients [57,58], though data specific to AKI patients was lacking. Hypocaloric nutrition is suggested at the early phase of critical illness to avoid overfeeding as substantial energy is produced endogenously via substrate mobilization [59]. In preclinical studies, energy restriction has been shown to exert renoprotective effects during AKI [60]. Although a greater amount of protein provision is recommended to achieve positive nitrogen balance, particularly in AKI patients undergoing KRTs [56], high protein intakes are potentially associated with negative metabolic complications and poorer clinical outcomes [61,62]. Future work is required to determine optimal energy and protein provision to patients with AKI. 

The present systematic review has several limitations. Firstly, this systematic review included observational studies, which should be interpreted as an association and do not necessarily imply causality. Secondly, meta-analyses of individual parameters of PEW cannot be performed due to the heterogeneity in outcomes reported (in-hospital mortality, 28-day mortality, or 90-day mortality, etc.) and expressed (OR, RR, or HR). Thirdly, some studies were not designed to determine the prognostic ability of these nutritional parameters as the primary objective, therefore their results should be interpreted with caution. In addition, these studies recruited patients from hospital or ICU, therefore the findings should not be generalized to patients with community-acquired AKI. Lastly, we also cannot determine the association of PEW criteria with other clinical outcomes such as ICU or hospital length of stay, renal recovery, quality of life, and physical function due to lack of studies that reported these outcomes. 

## 5. Conclusions

SGA is a valid tool for assessment of PEW in patients with AKI, as it has been demonstrated to be associated with increased mortality risk. Individual nutrition parameters for PEW assessment in isolation did not have consistent prognostic validity with clinical outcomes in patients with AKI. Serum proteins by themselves are not reliable nutrition markers for guiding and monitoring nutrition prescription in AKI patients with critical illness. On the other hand, there is a critical need for further experimental investigations on the body and muscle mass criteria to assess PEW in patients with AKI. Further studies are also warranted to examine whether AKI patients diagnosed with PEW will be benefited from aggressive nutrition therapy.

## Figures and Tables

**Table 1:** Summary of studies included in the review.

Author, Year	Country	Population	*n*	Male, *n* (%)	Age (Year)	SCr (μmol/L)	Sepsis (%)	KRT (%)	Mortality Rate (%)	PEW and Nutrition Parameter	Clinical Outcome
Bellomo 2014 [19,20]	Australia, New Zealand	Critical ill	1457	941 (64.6)	65.1	335	49.3	100	44.6	Energy intake, protein intake	28-day mortality, 90-day mortality, KRT-free day, ICU-free day, hospital-free day
Berbel, 2014 [21]	Brazil	ATN	133	91 (68.4)	61.1	359	31.0	57.8	44.5	Albumin, total cholesterol, arm circumference, energy intake, SGA	In-hospital mortality
Bufarah, 2018 [22]	Brazil	ATN	595	384 (64.5)	64.0	301	NA	52.0	46.0	Albumin, total cholesterol, energy intake, protein intake	In-hospital mortality
Chertow, 1998 [23]	United States	Critical ill, ATN	256	166 (65.0)	62.0	407	30.0	20.0	36.3	Albumin	60-day mortality
de Goes, 2018 [24]	Brazil	Critical ill, ATN	124	86 (69.4)	61.1	389	47.6	100	73.0	Protein intake	28-day mortality
Demirjian, 2011 [25]	United States	Critical ill	321	209 (65.1)	59.0	362	NA	100	66.0	Albumin	28-day mortality
Fiaccadori, 1999 [26]	Italy	AKI patients in RICU	309	197 (63.8)	67.0	522	23.3	67.0	39.0	SGA	In-hospital mortality
Gong, 2012 [27]	China	Elderly (≥ 65 years old)	99	62 (62.6)	77.9	165	29.3	12.1	42.4	Pre-albumin	Mortality
Guimaraes, 2008 [28]	Brazil	Critical ill	56	36 (64.3)	58.1	292	64.3	23.2	69.6	Total cholesterol, SGA	28-day mortality
Kritmetapak, 2016 [29]	Thailand	Critical ill	70	47 (67.1)	60.7	316	NA	100	61.4	Protein intake, SGA	28-day mortality
Lin, 2009 [30]	Taiwan	Critical ill, post-operative	342	204 (59.6)	64.0	292	NA	100	59.9	Body mass index	90-day mortality
Lins, 2000 [31]	Belgium	Critical ill	197	119 (60.4)	69.8	380	NA	26.0	53.0	Albumin	In-hospital mortality
Mendu, 2017 [32]	United States	Critical ill	176	102 (58.0)	61.0	301	NA	49.0	50.0	Albumin	60-day mortality
Sezer, 2008 [33]	Turkey	Critical ill	64	34 (53.1)	63.7	NA	NA	20.0	50.0	Albumin	Mortality
Wang, 2017 [34]	China	Hospital acquired AKI	340	247 (72.6)	62.5	158	17.1	13.8	27.6	Prealbumin	90-day mortality
Xie, 2011 [35]	China	Hospital acquired AKI	155	115 (74.2)	63.4	165	43.8	19.4	33.5	Albumin, prealbumin, total cholesterol	90-day mortality

AKI, acute kidney injury; ATN, acute tubular necrosis; ICU, intensive care unit; KRT, kidney replacement therapy; NA, not available; PEW, protein-energy wasting; RICU, renal intermediate care unit; SCr, serum creatinine; SGA, subjective global assessment.

**Table 2:** Nutrition parameters and clinical outcomes.

Study	Mean ± SD/Median (IQR)	Results
Serum chemistry		
*Serum albumin*		
Sezer, 2008 [33]	3.2 ± 0.8 g/dL	Serum albumin was not associated with mortality (β = 0.247, 95% CI: 0.047–1.304, *p* = 0.100)
Berbel, 2014 [21]	2.4 g/dL *	Serum albumin was not associated with in-hospital mortality (OR: 0.436, 95% CI: 0.124–1.528, *p* = 0.19)
Bufarah, 2018 [22]	2.4 g/dL *	Higher serum albumin was associated with lower in-hospital mortality (OR: 0.545, 95% CI: 0.401–0.417, *p* < 0.001)
Lins, 2000 [31]	3.2 ± 0.9 g/dL	Lower serum albumin was associated with higher in-hospital mortality (RR: 1.50, 95% CI: 1.14–1.97)
Demirjian, 2011 [25]	2.4 ± 0.7 g/dL	Higher serum albumin was associated with lower 28-day mortality (HR: 0.76, 95% CI: 0.59–0.98, *p* = 0.04)
Mendu, 2017 [32]	2.5 ± 0.6 g/dL	Higher serum albumin was associated with lower 60-day mortality (OR: 0.49, 95% CI: 0.27–0.89, *p* = 0.02)
Chertow, 1998 [23]	2.7 ± 0.7 g/dL	Serum albumin (per g/dL) was not associated with 60-day mortality (RR: 0.73, 95% CI: 0.51–1.04, *p* = 0.08)
Xie, 2011 [35]	3.2 ± 0.7 g/dL	Serum albumin (per 0.5 g/dL decrease) was not associated with 90-day mortality (HR: 0.967, *p* = 0.737)
*Serum prealbumin*		
Gong, 2012 [27]	13.5 (7.7) mg/dL	Serum prealbumin was not associated with mortality (OR: 0.328, 95% CI: 0.095–1.135, *p* = 0.078)
Wang, 2017 [34]	17.6 ± 6.9 mg/dL	Serum prealbumin <10 mg/dL was associated with greater 90-day mortality (HR: 2.55, 95% CI: 1.18–5.49, *p* = 0.02)
Xie, 2011 [35]	15.1 ± 6.8 mg/dL	Serum prealbumin (per 5 mg/dL decrease) was not associated with 90-day mortality (HR: 1.099, *p* = 0.414)
*Serum total cholesterol*		
Berbel, 2014 [21]	125 mg/dL *	Serum total cholesterol was not associated with in-hospital mortality (OR: 1.005, 95% CI: 0.997–1.013, *p* = 0.19)
Burfarah, 2008 [22]	119 mg/dL *	Serum total cholesterol was not associated with in-hospital mortality (OR: 0.995, 95% CI: 0.991–1.000, *p* = 0.052)
Guimaraes, 2008 [28]	101 ± 52 mg/dL	Serum total cholesterol ≤ 96 mg/dL was associated with higher 28-day mortality (HR: 10.94, 95% CI: 1.89–63.29, *p* = 0.008)
Xie, 2011 [35]	139 ± 58 mg/dL	A decrease of 3 mg/dL in serum total cholesterol was not associated with 90-day mortality (HR: 0.949, *p* = 0.470)
Body mass		
*Body mass index*		
Lin, 2009 [30]	23.5 ± 3.8 kg/m^2^	Higher body mass index was associated with lower mortality (OR: 0.903, 95% CI 0.840–0.971, *p* = 0.006).
Muscle mass		
*Arm circumference*		
Berbel, 2014 [21]	29.9 ± 5.4 cm *	Arm circumference was not associated with in-hospital mortality (OR: 0.961; 95% CI: 0.850–1.086, *p* = 0.52)
Dietary intake		
*Energy intake*		
Bellomo, 2014 [19]	11.0 ± 9.0 kcal/kg	Energy intake was not associated with 90-day mortality (OR: 1.079, 95% CI: 0.55–2.13, *p* = 0.8275), KRT free days (*p* = 0.2695), ICU-free days (*p* = 0.4714), and hospital free days (*p* = 0.5625).
Berbel, 2014 [21]	12.1 kcal/kg *	Higher energy intake was associated with lower in-hospital mortality (OR: 0.950, 95% CI: 0.910–0.991, *p* = 0.020)
Bufarah, 2018 [22]	13.5 kcal/kg *	Higher energy intake was associated with lower in-hospital mortality (OR: 0.946, 95% CI: 0.901–0.994; *p* = 0.029)
*Protein intake*		
Bellomo, 2014 [20]	0.50 ± 0.40 g/kg	Protein intake was not associated with 90-day mortality (OR: 0.998, 95% CI: 0.99–1.01, *p* = 0.6413), KRT-free days (*p* = 0.5792), MV-free days (*p* = 0.7564), ICU-free days (*p* = 0.6801), and hospital-free days (*p* = 0.5991)
Bufarah, 2018 [22]	0.64 g/kg *	Higher protein intake was associated with lower in-hospital mortality (OR: 0.947; 95% CI: 0.988–0.992; *p* = 0.028)
de Goes, 2018 [24]	31.5 g *	Higher protein intake was associated with lower 28-day mortality (HR: 0.993, 95% CI: 0.987–0.999, *p* = 0.032)
Kritmetapak, 2016 [29]	0.62 ± 0.30 g/kg	Higher protein intake (per 0.2 g/kg) was associated with greater survival at day-28 (OR: 4.62; 95% CI: 1.48–14.47; *p* = 0.009)

CI, confidence interval; ICU, intensive care unit; IQR, interquartile range; HR, hazard ratio; KRT, kidney replacement therapy; MV, mechanical ventilation; OR, odds ratio; RR, risk ratio. * Means were estimated based on the median and interquartile ranged reported as by Wan et al. [36].

**Table 3:** Prevalence of PEW in patients with AKI based on SGA.

Study	Setting	*n*	Subjective Global Assessment (%)		
			Well-Nourished	Mildly/Moderately Malnourished	Severely Malnourished
Fiaccadori, 1999 [26]	RICU	309	42.1	16.2	41.7
Berbel, 2014 [21]	ICU and wards	133	39.8	42.9	17.3
Guimaraes, 2008 [28]	ICU	56	17.9	67.8	14.3
Kritmetapak, 2016 [29]	ICU	70	41.4	41.4	17.2

Abbreviations: AKI, acute kidney injury; ICU, intensive care unit; PEW, protein energy wasting; RICU, renal intermediate care unit; SGA, subjective global assessment.

## Supplementary Materials

The following are available online at https://www.mdpi.com/2072-6643/12/9/2809/s1, Table S1: Full search strategy, Table S2: Protein-energy wasting diagnostic criteria, Table S3: Study selection based on inclusion criteria after reviewing full text, Table S4: Summary of studies included in the review (additional information), Table S5: Quality assessment of studies included in the review, Figure S1: Sensitivity analysis, Figure S2: Funnel plot.
