# Supplementary material for: Protein-Energy Wasting Assessment and Clinical Outcomes in Patients with Acute Kidney Injury: A Systematic Review with Meta-Analysis
Source: Nutrients. 2020 Sep 13;12(9):2809. doi: 10.3390/nu12092809 (PMC7551057; doi:10.3390/nu12092809)
Supplement: Supplementary file 1 [file nutrients-12-02809-s001.pdf]

Table S1. Full search strategy

No.	Database	Search Terms and Results
1.	PubMed (12 Feb 2020)	#1: “Acute Kidney Injury”[Mesh] OR “Kidney/Injuries”[Mesh] OR “Continuous Renal Replacement Therapy”[Mesh] OR acute kidney injury[title/abstract] OR acute renal failure[title/abstract] OR continuous renal replacement therapy [title/abstract] **68380**<br>#2: “Nutrition Assessment”[Mesh] OR “Nutritional Status”[Mesh] OR “Protein-Energy Malnutrition”[Mesh] OR “Malnutrition”[Mesh] OR nutrition assessment [title/abstract] OR nutritional assessment[title/abstract] OR nutrition risk[title/abstract] OR nutritional risk[title/abstract] OR nutrition assessment[title/abstract] OR nutritional assessment[title/abstract] OR malnutrition[title/abstract] OR protein energy malnutrition[title/abstract] OR protein energy wasting[title/abstract] **186295**<br>#3: Anthropometry[Mesh] OR “Body Composition”[Mesh] OR “Muscle Strength”[Mesh] OR “Body Weights and Measures”[Mesh] OR body weight[title/abstract] OR body mass[title/abstract] OR body mass index[title/abstract] OR muscle mass[title/abstract] OR body fat[title/abstract] **925826**<br>#4: “Proteins/blood”[Mesh] OR albumin[title/abstract] OR prealbumin[title/abstract] OR cholesterol[title/abstract] **847985**<br>#5: “Nutrients”[Mesh] OR “Dietary Proteins”[Mesh] OR “Enteral Nutrition”[Mesh] OR “Parenteral Nutrition”[Mesh] OR energy intake[title/abstract] OR protein intake[title/abstract] OR macronutrient*[title/abstract] OR micronutrient*[title/abstract] OR trace element*[title/abstract] **228498**<br>#6: #2 OR #3 OR #4 OR #5 **1978969**<br>#7: #1 AND #6 **6537**<br>#8: #7 NOT (“Animals”[Mesh] NOT “Humans”[Mesh]) **5296**<br>#9: #8 NOT ((“Infant”[Mesh] OR “Child”[Mesh] OR “Adolescent”[Mesh]) NOT “Adult”[Mesh]) **4802**<br>#10: #9 NOT (“Bibliography”[Publication Type] OR “Case Reports”[Publication Type] OR “Congress”[Publication Type] OR “Editorial”[Publication Type] OR “Guideline”[Publication Type] OR “Letter”[Publication Type] OR “Practice Guideline”[Publication Type] OR “Review”[Publication Type] OR “Technical Report”[Publication Type]) **3261**<br>#11: Filter: English **2955**
2.	Scopus (12 Feb 2020)	#1: INDEXTERMS(“Acute Kidney Injury” OR “Kidney/Injuries” OR “Continuous Renal Replacement Therapy”) OR TITLE-ABS(“acute kidney injury” OR “acute renal failure” OR “continuous renal replacement therapy”) **77415**<br>#2: INDEXTERMS(“Nutrition Assessment” OR “Nutritional Status” OR “Protein-Energy Malnutrition” OR “Protein Energy Wasting” OR Malnutrition) OR TITLE-ABS(“nutrition assessment” OR “nutrition status” OR “nutritional status” OR “nutrition risk” OR “nutritional risk” OR malnutrition OR “protein energy wasting” OR “protein-energy malnutrition”) **154914**

		#3: INDEXTERMS(Anthropometry OR “Body Composition” OR “Muscle Strength” OR “Body Weights and Measures”) OR TITLE-ABS(“body weight” OR “body mass” OR “body mass index” OR “muscle mass” OR “body fat”) **649821**<br>#4: INDEXTERMS(“Blood Proteins”) OR TITLE-ABS(albumin OR prealbumin OR cholesterol) **547401**<br>#5: INDEXTERMS(Nutrients OR “Dietary Proteins” OR “Enteral Nutrition” OR “Parenteral Nutrition”) OR TITLE-ABS(“energy intake” OR “protein intake” OR macronutrient* OR micronutrient* OR trace element*) **307685**<br>#6: #2 OR #3 OR #4 OR #5 **1535825**<br>#7: #1 AND #6 **5891**<br>#8: INDEXTERMS(Infant OR Child OR Adolescent) **3875741**<br>#9: #7 AND NOT #8 **5262**<br>#10: KEY (nonhuman OR animal OR “animal experiment”) **8586541**<br>#11: #9 AND NOT #10 **2866**<br>#12: DOCTYPE(ab OR bk OR ch OR bz OR cp OR cr OR dp OR ed OR er OR le OR no OR pr OR rp OR re OR sh)[†] **21779001**<br>#13: #11 AND NOT #12 **2304**<br>#14: #13 Limit to: English **2030**<br><br>[†]ab-abstract report; bk-book; ch-book chapter; bz-business article; cp-conference paper; cr-conference review; dp-data paper; ed-editorial; er-erratum; le-letter; no-note; pr-press release; rp-report; re-review; sh-short survey
3.	Cochrane (12 Feb 2020)	#1: MeSH descriptor: [Acute Kidney Injury] explode all trees **1378**<br>#2: "acute kidney injury" OR "acute renal failure" OR "continuous renal replacement therapy" **4456**<br>#3: #1 OR #2 **4483**<br>#4: MeSH descriptor: [Nutrition Assessment] explode all trees **681**<br>#5: MeSH descriptor: [Nutritional Status] explode all trees **2377**<br>#6: MeSH descriptor: [Protein-Energy Malnutrition] explode all trees **248**<br>#7: MeSH descriptor: [Malnutrition] explode all trees **4067**<br>#8: "nutrition assessment" OR "nutritional status" OR "nutrition status" OR "nutritional risk" OR "nutrition risk" OR "protein-energy malnutrition" OR "protein-energy wasting" **7713**<br>#9: #4 OR #5 OR #6 OR #7 OR #8 **10848**<br>#10: MeSH descriptor: [Anthropometry] explode all trees **22817**<br>#11: MeSH descriptor: [Body Composition] explode all trees **4878**<br>#12: MeSH descriptor: [Muscle Strength] explode all trees **5298**<br>#13: MeSH descriptor: [Body Weights and Measures] explode all trees **30943**<br>#14: "body weight" OR "body mass" OR "Body Mass Index" OR "muscle mass" OR "body fat" **90099**<br>#15: #10 OR #11 OR #12 OR #13 OR #14 **106209**<br>#16: MeSH descriptor: [Blood Proteins] explode all trees **51658**<br>#17: MeSH descriptor: [Albumins] explode all trees **7669**

	#18: MeSH descriptor: [Prealbumin] explode all trees **166**<br>#19: MeSH descriptor: [Cholesterol] explode all trees **10017**<br>#20: "albumin" OR "prealbumin" OR "cholesterol" **50159**<br>#21: #16 OR #17 OR #18 #19 OR #20 **96080**<br>#22: MeSH descriptor: [Nutrients] explode all trees **4941**<br>#23: MeSH descriptor: [Dietary Proteins] explode all trees **3943**<br>#24: MeSH descriptor: [Enteral Nutrition] explode all trees **1772**<br>#25: MeSH descriptor: [Parenteral Nutrition] explode all trees **1625**<br>#26: "energy intake" OR "protein intake" OR macronutrient* OR micronutrient* OR "trace element*" **14441**<br>#27: #22 OR #23 OR #24 OR #25 OR #26 **23021**<br>#28: #9 OR #15 OR #21 OR #27 **200784**<br>#29: #3 AND #28 **702**<br>#30: MeSH descriptor: [Infant] explode all trees **15806**<br>#31: MeSH descriptor: [Child] explode all trees **1215**<br>#32: MeSH descriptor: [Adolescent] explode all trees **102290**<br>#33: #30 OR #31 OR #32 **278658**<br>#34: #29 NOT #33 **562**†<br><br>†Cochrane Reviews = 29, Cochrane Protocol = 4, Trials = 528, Clinical Answers = 1

Table S2. Protein energy wasting diagnostic criteria

Criteria
Serum chemistry
Serum albumin < 3.8 g/dL
Serum prealbumin < 30 mg/dL
Serum cholesterol < 100 mg/dL
Body mass
Body mass index < 23 kg/m^2
Unintentional weight loss over time: 5% over 3 months or 10% over 6 months
Total body fat percentage <10%
Muscle mass
Muscle wasting: reduced muscle mass 5% over 3 months or 10% over 6 months
Reduced mid0arm muscle circumference area (reduction >10% in relation to 50th percentile of reference population)
Creatinine appearance
Dietary intake
Unintentional low dietary protein intake < 0.80 g/kg//day for at least 2 months
Unintentional low dietary energy intake < 25 kcal/kg/day

Table S3. Study selection based on inclusion criteria after reviewing full text

First author, year	Journal	Tittle	Include?	Reason of Exclusion
Abel, 1973	New England Journal of Medicine	Improved survival from acute renal failure after treatment with intravenous essential L-amino acids and glucose	No	No analysis on clinical outcomes based on PEW
Ahlstrom, 2004	Clinical Nephrology	Predictive value of interleukin 6,8 and 10 and low HLA-DR expression in acute renal failure	No	Not PEW
Allegretti, 2016	Digestive Diseases and Sciences	Siglec-7 as a Novel Biomarker to Predict Mortality in Decompensated Cirrhosis and Acute Kidney Injury	No	No analysis on clinical outcomes based on PEW
Alves, 2013	Nephrology Dialysis Transplantation	Hypomagnesemia as a risk factor for the non-recovery of the renal function in critically ill patients with acute kidney injury	No	Not PEW
Bagshaw, 2013	Journal of Critical Care	Urine biochemistry in septic and non-septic acute kidney injury: a prospective observational study	No	Not PEW
Basi, 2005	American Journal of Physiology-Renal Physiology	Insulin resistance in critically ill patients with acute renal failure	No	Not PEW
Bell, 2009	Nephrology Dialysis Transplantation	Cystatin C is correlated with mortality in patients with and without acute kidney injury	No	Not PEW
Bellomo, 1997	Renal Failure	A Prospective Comparative Study of Moderate Versus High Protein Intake for Critically Ill Patients with Acute Renal Failure	No	No clinical outcome
Bellomo, 2014	Critical Care	Calorie intake and patient outcomes in severe acute kidney injury: findings from The Randomized Evaluation of Normal vs. Augmented Level of Replacement Therapy (RENAL) study trial	Yes	
Bellomo, 2014	Blood Purification	Daily Protein Intake and Patient Outcomes in Severe Acute Kidney Injury: Findings of the Randomized Evaluation of Normal versus Augmented Level of Replacement Therapy (RENAL) Trial	Yes	
Berbel, 2014	Clinics	Nutritional parameters are associated with mortality in acute kidney injury	Yes	
Biagioni Santos, 2010	Brazilian Journal of Medical and Biological Research	Hypomagnesemia is a risk factor for nonrecovery of renal function and mortality in AIDS patients with acute kidney injury	No	Not PEW
Bouchard, 2009	Kidney International	Fluid accumulation, survival and recovery of kidney function in critically ill patients with acute kidney injury	No	No analysis on clinical outcomes based on PEW

First author, year	Journal	Tittle	Include?	Reason of Exclusion
Bufarah, 2018	Clinical Nutrition ESPEN	Low caloric and protein intake is associated with mortality in patients with acute kidney injury	Yes	
Cerda, 2009	Nephrology Dialysis Transplantation	In severe acute kidney injury, a higher serum creatinine is paradoxically associated with better patient survival	No	Not a full length article
Chen, 2012	PLoS One	Acute Kidney Injury Biomarkers for Patients in a Coronary Care Unit: A Prospective Cohort Study	No	Non-AKI population
Chen, 2015	Critical Care	Fluid overload at start of continuous renal replacement therapy is associated with poorer clinical condition and outcome: a prospective observational study on the combined use of bioimpedance vector analysis and serum N-terminal pro-B-type natriuretic peptide measurement	No	Not PEW
Chewtow, 1998	Journal of American Society of Nephrology	Predictors of mortality and the provision of dialysis in patients with acute tubular necrosis	Yes	
Chuasuwan, 2006	Journal of the Medical Association of Thailand	Continuous Veno-Venous Hemofiltration in Bhumibol Adulyadej Hospital	No	Retrospective study
Coca, 2014	Journal of American Society of Nephrology	Urinary Biomarkers of AKI and Mortality 3 Years after Cardiac Surgery	No	Not PEW
Costa, 2018	Bioscience Reports	Protein carbonyl concentration as a biomarker for development and mortality in sepsis-induced acute kidney injury	No	Not PEW
Danziger, 2016	Critical Care Medicine	Obesity, Acute Kidney Injury, and Mortality in Critical Illness	No	Retrospective study
de Souza, 2014	Jornal Brasileiro de Nefrologia	Inverse association between serum creatinine and mortality in acute kidney injury	No	Not PEW
Demirjian, 2011	Nephrology Dialysis Transplantation	Hypophosphatemia during continuous hemodialysis is associated with prolonged respiratory failure in patients with acute kidney injury	Yes	
Demirjian, 2011	Clinical Journal of American Society of Nephrology	Model to Predict Mortality in Critically Ill Adults with Acute Kidney Injury	No	No analysis on clinical outcomes based on PEW
Dharan, 2005	Renal Failure	Prediction of Mortality in Acute Renal Failure in the Tropics	No	Doubtful results
Dimitrijevic,	The Tohoku Journal of	Elevated Serum Ferritin Levels Are Predictive of Renal Function	No	Not PEW

First author, year	Journal	Tittle	Include?	Reason of Exclusion
2019	Experimental Medicine	Recovery among Patients with Acute Kidney Injury		
Doig, 2015	Intensive Care Medicine	Intravenous amino acid therapy for kidney function in critically ill patients: a randomized controlled trial	No	No analysis on clinical outcomes based on PEW
Druml, 2010	Intensive Care Medicine	Impact of body mass on incidence and prognosis of acute kidney injury requiring renal replacement therapy	No	Retrospective study
Feinstein, 1981	Medicine	Clinical and metabolic responses to parenteral nutrition in acute renal failure	No	No analysis on clinical outcomes based on PEW
Fiaccadori, 1999	Journal of American Society of Nephrology	Prevalence and Clinical Outcome Associated with Preexisting Malnutrition in Acute Renal Failure: A Prospective Cohort Study	Yes	
Ficek, 2006	Kidney & Blood Pressure Research	Plasma Concentrations of Tumor Necrosis Factor Alpha May Predict the Outcome of Patients with Acute Renal Failure	No	No analysis on clinical outcomes based on PEW
Franklin, 1997	American Journal of Physiology-Renal Physiology	Insulin-like Growth Factor I Preserves Renal Function Postoperatively	No	Not PEW
Fulop, 2010	ASAIO Journal	Volume-Related Weight Gain and Subsequent Mortality in Acute Renal Failure Patients Treated With Continuous Renal Replacement Therapy	No	No analysis on clinical outcomes based on PEW
de Goes, 2018	Nutrients	Evaluation of Factors Associated with Hypermetabolism and Hypometabolism in Critically Ill AKI Patients	Yes	
Gong, 2012	Archives of Gerontology and Geriatrics	Elderly patients with acute kidney injury (AKI): Clinical features and risk factors for mortality	Yes	
Gong, 2019	Clinical Interventions in Aging	Can Serum Nutritional Related Biomarkers Predict Mortality Of Critically Ill Older Patients With Acute Kidney Injury?	No	No analysis on clinical outcomes based on PEW
Guimaraes, 2008	Critical Care Medicine	Low insulin-like growth factor-1 and hypocholesterolemia as mortality predictors in acute kidney injury in the intensive care unit	Yes	
Gunay, 2018	Northern Clinics of Istanbul	Increase of endocan, a new marker for inflammation and endothelial dysfunction, in acute kidney injury	No	No analysis on clinical outcomes based on PEW
Gunstm 2013	Journal of American Society of Nephrology	Impact of Early Parenteral Nutrition on Metabolism and Kidney Injury	No	No analysis on clinical outcomes based on PEW

First author, year	Journal	Tittle	Include?	Reason of Exclusion
Hall, 2018	BMC Nephrology	suPAR as a marker of infection in acute kidney injury – a prospective observational study	No	Not PEW
Hamzic-Mehmedbasic, 2016	Journal of Renal Injury Prevention	Prognostic indicators of adverse renal outcome and death in acute kidney injury hospital survivors	No	No analysis on clinical outcomes based on PEW
Han, 2014	Journal of Critical Care	Mean platelet volume is a prognostic factor in patients with acute kidney injury requiring continuous renal replacement therapy	No	Retrospective study
Hsu, 2020	JAMA Internal Medicine	Post–Acute Kidney Injury Proteinuria and Subsequent Kidney Disease Progression The Assessment, Serial Evaluation, and Subsequent Sequelae in Acute Kidney Injury (ASSESS-AKI) Study	No	Not PEW
Huang, 2013	PLoS One	Procalcitonin Levels Predict Acute Kidney Injury and Prognosis in Acute Pancreatitis: A Prospective Study	No	Not PEW
Huelin, 2019	Hepatology	Neutrophil Gelatinase-Associated Lipocalin for Assessment of Acute Kidney Injury in Cirrhosis: A Prospective Study	No	No analysis on clinical outcomes based on PEW
Iglesias, 2003	American Journal of Kidney Disease	Elevated Serum Levels of the Type I and Type II Receptors for Tumor Necrosis Factor-α as Predictive Factors for ARF in Patients With Septic Shock	No	No analysis on clinical outcomes based on PEW
Jaques, 2018	Nephrology	Biomarkers for acute kidney injury in decompensated cirrhosis: a retrospective study	No	Not PEW
Jeong, 2013	Renal Failure	Role of B-type natriuretic peptide as a marker of mortality in acute kidney injury patients treated with continuous renal replacement therapy	No	No analysis on clinical outcomes based on PEW
Jung, 2016	Medicine	Electrolyte and mineral disturbances in septic acute kidney injury patients undergoing continuous renal replacement therapy	No	Not PEW
Jung, 2018	PLoS One	Phosphate is a potential biomarker of disease severity and predicts adverse outcomes in acute kidney injury patients undergoing continuous renal replacement therapy	No	Not PEW
Kadiroglu, 2007	Renal Failure	The Evaluation of Effects of Demographic Features, Biochemical Parameters, and Cytokines on Clinical Outcomes in Patients with Acute Renal Failure	No	No analysis on clinical outcomes based on PEW
Kim, 2015	Journal of Nutrition	The effect of nutritional supply on clinical outcomes and nutritional status	No	Non English language

First author, year	Journal	Tittle	Include?	Reason of Exclusion
	and Health	in critically ill patients receiving continuous renal replacement therapy		
Kim, 2017	Kidney Research and Clinical Practice	Body mass index is inversely associated with mortality in patients with acute kidney injury undergoing continuous renal replacement therapy	No	Retrospective study
Kim, 2017	PLoS One	Fluid overload and survival in critically ill patients with acute kidney injury receiving continuous renal replacement therapy	No	Retrospective study
Kim, 2018	Kidney & Blood Pressure Research	A Prospective Observational Study on the Predictive Value of Serum Cystatin C for Successful Weaning from Continuous Renal Replacement Therapy	No	Not PEW
Kim, 2018	BMC Nephrology	The impact of disease severity on paradoxical association between body mass index and mortality in patients with acute kidney injury undergoing continuous renal replacement therapy	No	Retrospective study
Kocyigit, 2013	Turkish Nephrology, Dialysis and Transplantation Journal	Trends in acute renal failure in central Anatolia	No	Inclusion of patients < 18 years old
Kohli, 2007	International Urology Nephrology	Predictors of mortality in elderly patients with acute renal failure in a developing country	No	Not PEW
Kolyada, 2009	Kidney International	A genetic variant of hypoxia-inducible factor-1a is associated with adverse outcomes in acute kidney injury	No	Not PEW
Kopple, 1982	Proc EDTA	Current problems in amino acid therapy for acute renal failure	No	No analysis on clinical outcomes based on PEW
Koyner, 2012	Journal of American Society of Nephrology	Biomarkers Predict Progression of Acute Kidney Injury after Cardiac Surgery	No	Not PEW
Koyner, 2015	Journal of American Society of Nephrology	Furosemide Stress Test and Biomarkers for the Prediction of AKI Severity	No	Not PEW
Kritmetapak, 2016	PLoS One	The Impact of Macro-and Micronutrients on Predicting Outcomes of Critically Ill Patients Requiring Continuous Renal Replacement Therapy	Yes	
Kumpers, 2010	Intensive Care Medicine	Angiopoietin-2 in patients requiring renal replacement therapy in the ICU: relation to acute kidney injury, multiple organ dysfunction syndrome and outcome	No	Not PEW

First author, year	Journal	Tittle	Include?	Reason of Exclusion
Kumpers, 2010	Critical Care	Serum neutrophil gelatinase-associated lipocalin at inception of renal replacement therapy predicts survival in critically ill patients with acute kidney injury	No	Not PEW
Kurtin, 1987	American Journal of Kidney Disease	Profound hypophosphatemia in the course of acute renal failure	No	Not PEW
Lai, 2013	PLoS One	Is the Serum Vitamin D Level at the Time of Hospital-Acquired Acute Kidney Injury Diagnosis Associated with Prognosis?	No	Not PEW
Leaf, 2013	Clinical Journal of American Society of Nephrology	FGF-23 Levels in Patients with AKI and Risk of Adverse Outcomes	No	Not PEW
Leaf, 2013	Clinical Endocrinology	Dysregulated mineral metabolism in patients with acute kidney injury and risk of adverse outcomes	No	Not PEW
Leaf, 2018	Clinical Journal of American Society of Nephrology	Fibroblast Growth Factor 23 Associates with Death in Critically Ill Patients	No	Not PEW
Leaf, 2019	Journal of American Society of Nephrology	Iron, Hepcidin, and Death in Human AKI	No	Not PEW
Leblanc, 1998	American Journal of Kidney Disease	Catabolism in Critical Illness: Estimation From Urea Nitrogen Appearance and Creatinine Production During Continuous Renal Replacement Therapy	No	No analysis on clinical outcomes based on PEW
Leonard, 1975	Urology	Parenteral essential amino acids in acute renal failure	No	Not PEW
Li, 2010	BMC Nephrology	Malnutrition and inflammation in acute kidney injury due to earthquake-related crush syndrome	No	No analysis on clinical outcomes based on PEW
Li, 2019	Aging Clinical and Experimental Research	Analysis of the short-term prognosis and risk factors of elderly acute kidney injury patients in different KDIGO diagnostic windows	No	Retrospective study
Lin, 2009	The American Journal of Surgery	The 90-day mortality and the subsequent renal recovery in critically ill surgical patients requiring acute renal replacement therapy	Yes	
Lin, 2013	PLoS One	Serum Interleukin-18 at Commencement of Renal Replacement Therapy Predicts Short-Term Prognosis in Critically Ill Patients with Acute Kidney Injury	No	No analysis on clinical outcomes based on PEW

First author, year	Journal	Tittle	Include?	Reason of Exclusion
Lin, 2019	Bosnian Journal of Basic Medical Sciences	Expression patterns and prognostic value of miR-210, miR-494, and miR-205 in middle-aged and old patients with sepsis-induced acute kidney injury	No	No analysis on clinical outcomes based on PEW
Lins, 2000	Clinical Nephrology	Prognostic value of a new scoring system for hospital mortality in acute renal failure	Yes	
Lins, 2004	Nephrology Dialysis Transplantation	Re-evaluation and modification of the Stuivenberg Hospital Acute Renal Failure (SHARF) scoring system for the prognosis of acute renal failure: an independent multicentre, prospective study	No	No analysis on clinical outcomes based on PEW
Liu, 2017	BMC Nephrology	Surfactant protein-D (SP-D) gene polymorphisms and serum level as predictors of susceptibility and prognosis of acute kidney injury in the Chinese population	No	Not PEW
Lorenzen, 2017	Nephrology Dialysis Transplantation	Osteopontin predicts survival in critically ill patients with acute kidney injury	No	No analysis on clinical outcomes based on PEW
Mahajan, 2006	Renal Failure	Spectrum of Acute Renal Failure and Factors Predicting Its Outcome in an Intensive Care Unit in India	No	No analysis on clinical outcomes based on PEW
Malbouisson, 2019	Einstein	Lipid profile and statin use in critical care setting: implications for kidney outcome	No	No analysis on clinical outcomes based on PEW
Mault, 1983	ASAIO Journal	Starvation: a major contribution to mortality in acute renal failure?	No	Not PEW
McMahon, 2019	American Journal of Nephrology	Biomarker Predictors of Adverse Acute Kidney Injury Outcomes in Critically Ill Patients: The Dublin Acute Biomarker Group Evaluation Study	No	Not PEW
Meersch, 2018	Journal of American Society of Nephrology	Long-Term Clinical Outcomes after Early Initiation of RRT in Critically Ill Patients with AKI	No	Not PEW
Mendu, 2017	Clinical Journal of American Society of Nephrology	A Decision-Making Algorithm for Initiation and Discontinuation of RRT in Severe AKI	Yes	
Murugan, 2014	Nephrology Dialysis Transplantation	Plasma inflammatory and apoptosis markers are associated with dialysis dependence and death among critically ill patients receiving renal replacement therapy	No	Not PEW
Murugan,	Clinical Journal of	Associations between Intensity of RRT, Inflammatory Mediators, and	No	Not PEW

First author, year	Journal	Tittle	Include?	Reason of Exclusion
2015	American Society of Nephrology	Outcomes		
Murugan, 2018	Critical Care	Net ultrafiltration intensity and mortality in critically ill patients with fluid overload	No	Not PEW
Murugan, 2019	JAMA Network Open	Association of Net Ultrafiltration Rate With Mortality Among Critically Ill Adults With Acute Kidney Injury Receiving Continuous Venovenous Hemodiafiltration A Secondary Analysis of the Randomized Evaluation of Normal vs Augmented Level (RENAL) of Renal Replacement Therapy Trial	No	Not PEW
Pedersen, 2017	Osteoporosis International	Impact of body mass index on risk of acute kidney injury and mortality in elderly patients undergoing hip fracture surgery	No	Retrospective study
Pike, 2015	Clinical Journal of American Society of Nephrology	Biomarker Enhanced Risk Prediction for Adverse Outcomes in Critically Ill Patients Receiving RRT	No	Not PEW
Prabhu, 2016	Clinical Nephrology	Fever, thrombocytopenia, and AKI-A profile of malaria, dengue, and leptospirosis with renal failure in a South Indian tertiary-care hospital	No	Not PEW
Qian, 2015	Kidney & Blood Pressure Research	Serum Protein Thiol Levels in Patients with Hospital-Acquired Acute Kidney Injury	No	Not PEW
Quinto, 2015	Cytokine	TNF-α depuration is a predictor of mortality in critically ill patients under continuous veno-venous hemodiafiltration treatment	No	Not PEW
Sakan, 2018	Therapeutic Apheresis and Dialysis	Consequence of Elevated Fibroblast Growth Factor 23 Levels in Acute Kidney Injury, Renal Recovery and Overall Survival in Intensive Care Unit Patients After Major Surgery	No	Not PEW
Salahudeen, 2009	Clinical Journal of American Society of Nephrology	Sustained Low Efficiency Dialysis in the Continuous Mode (C-SLED): Dialysis Efficacy, Clinical Outcomes, and Survival Predictors in Critically Ill Cancer Patients	No	Retrospective study
Saly, 2017	PLoS One	Approaches to Predicting Outcomes in Patients with Acute Kidney Injury	No	Not PEW
Scheinkestel, 2003	Nutrition	Prospective Randomized Trial to Assess Caloric and Protein Needs of Critically Ill, Anuric, Ventilated Patients Requiring Continuous Renal Replacement Therapy	No	Non-AKI population

First author, year	Journal	Tittle	Include?	Reason of Exclusion
Sever, 2002	Nephrology Dialysis Transplantation	The Marmara earthquake: admission laboratory features of patients with nephrological problems	No	No analysis on clinical outcomes based on PEW
Sezer, 2006	Acta Medica	Predictors of mortality in patients with acute renal failure	No	Age range: 15-90 years old
Sezer, 2008	Journal of Renal Nutrition	Relevance of Nutritional Route and Intercellular Adhesion Molecule-1 in Patients With Acute Renal Failure and Its Prognostic Implications	Yes	
Shiao, 2017	PLoS One	Perioperative body weight change is associated with in-hospital mortality in cardiac surgical patients with postoperative acute kidney injury	No	Not PEW
Shum, 2016	Renal Failure	Septic acute kidney injury in critically ill patients – a single-center study on its incidence, clinical characteristics, and outcome predictors	No	Retrospective study
Simmons, 2004	Kidney International	Plasma cytokine levels predict mortality in patients with acute renal failure	No	Not PEW
Sole, 2019	Liver International	Characterization of inflammatory response in hepatorenal syndrome: Relationship with kidney outcome and survival	No	Not PEW
Srisawat, 2011	Kidney International	Plasma neutrophil gelatinase-associated lipocalin predicts recovery from acute kidney injury following community-acquired pneumonia	No	Not PEW
Stads, 2018	PLoS One	Fluid balance-adjusted creatinine at initiation of continuous venovenous hemofiltration and mortality. A post-hoc analysis of a multicenter randomized controlled trial	No	Not PEW
Valdivieso, 2008	Journal of Renal Nutrition	Impact of Prealbumin Levels on Mortality in Patients With Acute Kidney Injury: An Observational Cohort Study	No	Age range: 16-90 years old
Van Den Noortgate, 2003	Nephrology Dialysis Transplantation	Outcome in a post-cardiac surgery population with acute renal failure requiring dialysis: does age make a difference?	No	Retrospective study
Wang, 2006	Renal Failure	Early Prognostic Factors in Patients with Acute Renal Failure Requiring Dialysis	No	No analysis on clinical outcomes based on PEW
Wang, 2017	Oncotarget	Effects of continuous renal replacement therapy on serum cytokines, neutrophil gelatinase-associated lipocalin, and prognosis in patients with severe acute kidney injury after cardiac surgery	No	Not PEW
Wang, 2017	Scientific Reports	Serum prealbumin and its changes over time are associated with mortality	Yes	

First author, year	Journal	Tittle	Include?	Reason of Exclusion
		in acute kidney injury		
Woodward, 2019	Critical Care Medicine	Fluid Overload Associates With Major Adverse Kidney Events in Critically Ill Patients With Acute Kidney Injury Requiring Continuous Renal Replacement Therapy	No	Retrospective study
Wu, 2019	Frontiers in Immunology	Effects of changes in the levels of damage-associated molecular patterns following continuous veno-venous hemofiltration therapy on outcomes in acute kidney injury patients with sepsis	No	Not PEW
Xavier, 2016	Clinical Nutrition ESPEN	Handgrip strength and weight predict long-term mortality in acute kidney injury patients	No	AKI survivors only
Xie, 2011	BMC Nephrology	The ratio of CRP to prealbumin levels predict mortality in patients with hospital-acquired acute kidney injury	Yes	
Yang, 2018	Artificial Organs	Predictive Factors Upon Discontinuation of Renal Replacement Therapy for Long-Term Chronic Dialysis and Death in Acute Kidney Injury Patients	No	Not PEW
You, 2018	Clinical Interventions in Aging	Association of prealbumin levels with contrast-induced acute kidney injury in elderly patients with elective percutaneous coronary intervention	No	Non-AKI population
Zarbock, 2016	JAMA	Effect of Early vs Delayed Initiation of Renal Replacement Therapy on Mortality in Critically Ill Patients With Acute Kidney Injury The ELAIN Randomized Clinical Trial	No	Not PEW
Zhang, 2017	Journal of Investigative Medicine	Nutritional status plays a crucial role in the mortality of critically ill patients with acute renal failure	No	Retrospective study
Zheng, 2014	BioMed Research International	Metabolic Acidosis and Strong Ion Gap in Critically Ill Patients with Acute Kidney Injury	No	No analysis on clinical outcomes based on PEW
Zhu, 2018	Critical Care Medicine	The Effect of IV Amino Acid Supplementation on Mortality in ICU Patients May Be Dependent on Kidney Function: Post Hoc Subgroup Analyses of a Multicenter Randomized Trial	No	Non-AKI population
Zou, 2018	Cardio Renal Medicine	Role of Body Mass Index in Acute Kidney Injury Patients after Cardiac Surgery	No	Retrospective study
Zou, 2019	Internal Medicine Journal	Serum Prealbumin is prognostic for All-cause Mortality in Patients with Community-acquired and Post-operative Acute Kidney Injury	No	Retrospective study

Table S4. Summary of studies included in the review (additional information)

Author, year	Diagnostic criteria of AKI	Stage of AKI	Primary cause of AKI	Type of KRT	Comorbidities	Disease severity score	Inflammatory markers
Bellomo 2014	Predefined criteria	NA	NA	CVVHDF	Sepsis: 49.3%	APACHE III: 102.5	NA
Berbel, 2014	AKIN	AKIN 1: 11%<br>AKIN 2: 43%<br>AKIN 3: 46%	Ischemic: 49.6%<br>Nephrotoxic: 15.8%<br>Mixed: 34.6%	HD: 41.4%<br>PD: 16.5%	Sepsis: 31%<br>DM: 21%<br>CKD: 23%<br>Oliguria: 22%	ATN-ISS: 0.46	CRP: 14.4 mg/dL
Bufarah, 2018	AKIN	NA	Ischemic: 55.3%<br>Nephrotoxic: 9.9%<br>Septic: 10.1%<br>Mixed: 24.7%	HD or HVPD	DM: 25.5%<br>CKD: 30.1%	NA	CRP: 15.8 mg/dL
Chertow, 1998	NA	NA	ATN: 100%	NA	Sepsis: 30%<br>Infection: 47%<br>DM: 28%	NA	NA
de Goes, 2018	KDIGO	Stage 3	Septic: 80%<br>Ischemic: 13%<br>Nephrotoxicity: 5%<br>Mixed: 2%	NA	Sepsis: 47.6%<br>CVD: 32.3%	ATN-ISS: 0.65	CRP: 26.5 mg/dL
Demirjian, 2011	NA	NA	ATN: 56.1%<br>Multifactorial: 35.2%<br>Other: 8.7%	CVVHD	CKD: 35.5%<br>DM: 14.6%	APACHE II: 24	NA
Fiaccadori, 1999	Predefined criteria	NA	Medical AKI: 73%<br>Surgical AKI: 27%	HD or CRRT	Sepsis: 23.3%<br>Oliguria: 66%<br>DM: 12%	APACHE II: 23.1	NA
Gong, 2012	RIFLE	Rc: 19.2%<br>Ic: 31.3%<br>Fc: 49.5%	Ischemic: 53.5%<br>Surgical: 33.3%<br>Septic: 10.1%<br>Nephrotoxicity: 3.0%	NA	Sepsis/ inflection: 29.3%<br>MODS: 38.4%	APACHE II: 17.0<br>ATN-ISS: 0.34	CRP: 6.9 mg/dL

						SOFA: 6	
Guimaraes, 2008	Predefined criteria	NA	Ischemic: 69.6%<br>Multifactorial: 28.6%<br>Nephrotoxicity: 1.8%	NA	Sepsis: 64.3%<br>Multi-organ failure: 60.7%<br>Oliguria: 32.1%	APACHE II: 21<br>SOFA: 9.1	NA
Kritmetapak, 2016	NA	NA	Surgical AKI: 55.3%<br>Septic: 54.3%<br>Ischemic: 40.0%<br>Nephrotoxic: 1.4%<br>Multifactorial: 4.3%	CRRT	DM: 31.4%<br>Liver disease: 27.1%<br>Malignancy: 24.3%	APACHE II: 20.4<br>SOFA: 10.9	CRP: 11.5 mg/dL
Lin, 2009	RIFLE	Rc: 8.5%<br>Ic: 9.1%<br>Fc: 82.5%	Shock: 65.7%<br>Sepsis: 36.4%<br>Contrast nephropathy: 2.3%	IHD: 29.2%<br>CRRT: 20.8%	CKD: 72.5%	APACHE II: 12.4<br>SOFA: 11.8	NA
Lins, 2000	Predefined criteria	NA	Medical other: 77%<br>Medical toxic: 15%<br>Surgical: 8%	NA	NA	NA	NA
Mendu, 2017	NA	NA	Hypotension: 58%<br>Sepsis: 51%<br>Prerenal azotemia: 30%	CVVH: 25%<br>HD: 9%<br>CVVH & HD: 15%	Malignancy: 39%	NA	NA
Sezer, 2008	Predefined criteria	NA	Ischemic ATN: 66%<br>Nephrotoxic ATN: 19%<br>Acute GN: 9%	NA	Infection: 75%	APACHE III: 64	NA
Wang, 2017	RIFLE	Rc: 60.0%<br>Ic: 22.6%<br>Fc: 17.4%	Pre-renal: 39.7%<br>Renal: 27.4%<br>Pre-renal & renal: 32.9%	CRRT	Sepsis: 17.1%<br>Heart failure: 17.9%<br>DM: 7.9%	APACHE II: 14	hsCRP: 7.0 mg/L
Xie, 2011	RIFLE	Rc: 45.8%<br>Ic: 24.5%<br>Fc: 29.7%	Ischemic: 42.6%<br>Multifactorial: 37.4%<br>Nephrotoxic: 20%	NA	Sepsis: 43.8%<br>DM: 16.9%<br>CKD: 6.8%	SOFA: 7	CRP: 6.6 mg/dL

Abbreviations: AKI, acute kidney injury; AKIN, Acute Kidney Injury Network; APACHE, acute physiologic assessment and chronic health evaluation; ATN-ISS, acute tubular necrosis-index severity score; CKD, chronic kidney disease; CRP, C-reactive protein; CRRT, continuous renal replacement therapy; CVD, cardiovascular disease; CVVH, continuous venovenous hemodialysis; CVVHDF, continuous venovenous hemodiafiltration; DM, diabetes mellitus; HD, hemodialysis; hsCRP, high sensitivity C-reactive protein; Fc, class Failure; HVPD, high volume peritoneal dialysis; Ic, class Injury; IHD, intermittent hemodialysis; KDIGO, Kidney Disease Improving Global Outcomes; KRT, kidney replacement therapy; NA, not available; Rc, class Risk; RIFLE, Risk, Injury, Failure, Loss, End Stage Renal Disease; SOFA, Sequential Organ Failure Assessment.

Table S5. Quality assessment of studies included in the review

Author, year	Representativeness of exposed cohort	Selection of non-exposed cohort	Ascertainment of exposure	Outcome absent at start of study	Comparability of cohorts	Assessment of outcome	Adequate duration of follow up	Completeness of follow up
Bellomo, 2014	1	1	1	1	2	1	1	1
Berbel, 2014	Unclear	1	1	1	2	1	Unclear	1
Bufarah, 2018	Unclear	1	1	1	1	1	Unclear	Unclear
Chertow, 1998	1	1	1	1	2	1	1	1
Demirjian, 2011	Unclear	1	1	1	1	1	1	1
Fiaccadori, 1999	1	1	1	1	2	1	1	1
de Goes, 2018	Unclear	1	1	1	1	1	1	1
Gong, 2012	Unclear	1	1	1	2	1	Unclear	1
Guimaraes, 2008	1	1	1	1	1	1	1	1
Kritmetapak, 2016	1	1	1	1	1	1	1	1
Lin, 2009	1	1	1	1	2	1	1	1
Lins, 2000	Unclear	1	1	1	1	1	Unclear	1
Mendu, 2017	Unclear	1	1	1	1	1	1	1
Sezer, 2008	Unclear	1	1	1	1	1	Unclear	1
Wang, 2017	1	1	1	1	2	1	1	1
Xie, 2011	Unclear	1	1	1	2	1	1	1

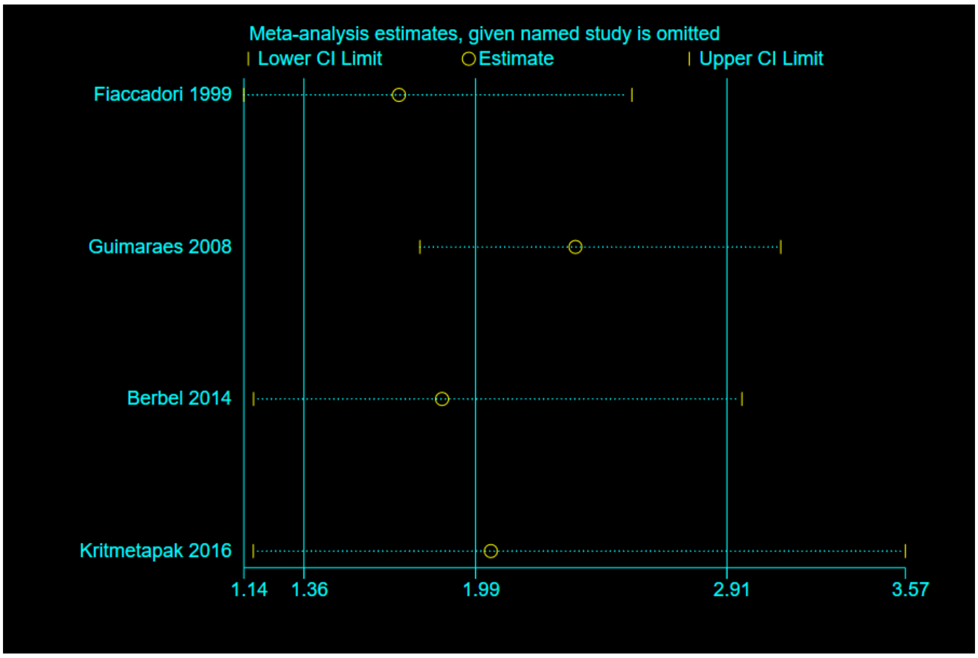

Figure S1: Sensitivity analysis

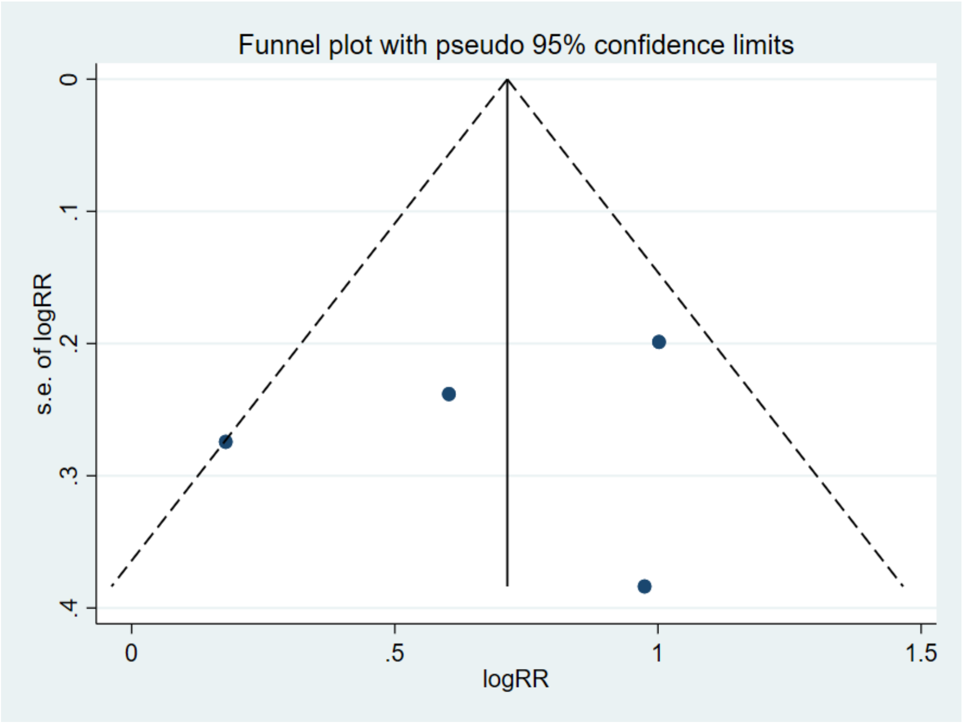

Figure S2: Funnel plot
